# A Pilot Study of the Role of Salivary Biomarkers in the Diagnosis of PCOS in Adolescents Across Different Body Weight Categories

**DOI:** 10.3390/jcm14176159

**Published:** 2025-08-31

**Authors:** Justyna Opydo-Szymaczek, Natalia Wendland, Dorota Formanowicz, Anna Blacha, Grażyna Jarząbek-Bielecka, Paulina Radomyska, Dominika Kruszyńska, Małgorzata Mizgier

**Affiliations:** 1 Chair and Department of Pediatric Dentistry, Poznan University of Medical Sciences, 70 Bukowska Street, 60-812 Poznan, Poland; nataliakryszan@interia.pl (N.W.); pradomyska@ump.edu.pl (P.R.); dominika.frontczak@gmail.com (D.K.); 2 Chair and Department of Medical Chemistry and Laboratory Medicine, Poznan University of Medical Sciences, 8 Rokietnicka Street, 60-806 Poznan, Poland; doforman@ump.edu.pl (D.F.); achlebna@ump.edu.pl (A.B.); 3 Chair and Department of Gynaecology, Poznan University of Medical Sciences, 22 Polna Street, 60-535 Poznan, Poland; grajarz@o2.pl; 4 Department of Sports Dietetics, Chair of Dietetics, Faculty of Health Sciences, Poznan University of Physical Education, Królowej Jadwigi 27/39, 61-871 Poznan, Poland; mizgier@awf.poznan.pl

**Keywords:** polycystic ovary syndrome, hyperandrogenism, adolescents, salivary assays, testosterone, TNF-α, IL-6, IL-1β, uric acid

## Abstract

**Background/Objectives**: Polycystic ovary syndrome (PCOS) is a complex endocrine disorder affecting reproductive, metabolic, and inflammatory processes in women of reproductive age. This study explored the diagnostic potential of salivary cytokines, uric acid, and testosterone in distinguishing PCOS patients from healthy controls, as well as to examine their associations with hormonal and metabolic profiles within the PCOS group. **Methods**: Forty-one adolescent girls with PCOS and thirty healthy controls participated in the study. The PCOS group included both normal-weight and overweight individuals, allowing evaluation of salivary biomarkers across different nutritional statuses. Salivary levels of TNF-α, IL-6, IL-1β, testosterone, and uric acid were measured and compared between the groups. A receiver operating characteristic (ROC) analysis was performed to assess the diagnostic value of each biomarker. **Results**: Salivary TNF-α, IL-6, and IL-1β showed high diagnostic accuracy (AUC = 0.921, 0.891, and 0.870, respectively), supporting their potential as non-invasive biomarkers. The diagnostic accuracy of salivary cytokines and testosterone remained high even in normal-weight participants, suggesting that low-grade inflammation and hormonal disturbances in PCOS are not limited to excess body weight. Salivary testosterone was strongly associated with hyperandrogenism, while uric acid correlated with the cortisol/DHEA-S ratio, indicating possible links to metabolic stress. **Conclusions**: In conclusion, salivary assays may offer a valuable, non-invasive tool for the early diagnosis of PCOS in adolescents, including normal-weight girls. This approach could facilitate the timely detection of inflammatory and hormonal imbalances, supporting earlier interventions and more personalized care.

## 1. Introduction

Polycystic ovary syndrome (PCOS) is a prevalent endocrine disorder that affects women of reproductive age. It is characterized by elevated androgen levels, irregular ovulation, and the presence of polycystic ovaries [1,2]. In addition to its impact on reproductive health, PCOS is often associated with metabolic disturbances such as insulin resistance [1,2]. Moreover, women with PCOS often face mental health challenges, including anxiety and depression, which can significantly affect their quality of life [3]. The syndrome has a complex pathophysiology, involving a combination of hormonal imbalances and inflammatory processes [1,4,5,6].

Research studies indicate that pro-inflammatory cytokines, such as interleukin-6 (IL-6), interleukin-1β (IL-1β), and tumor necrosis factor-α (TNF-α), are elevated in the saliva and blood of women with PCOS, reflecting a chronic low-grade inflammation state [5,7,8,9,10]. This inflammation exacerbates the metabolic dysfunction and hormonal imbalance observed in PCOS [5,10]. Furthermore, hyperandrogenism, a hallmark feature of PCOS, is closely linked to both the metabolic and reproductive abnormalities characteristic of the syndrome [1,6].

In addition to inflammation and hyperandrogenism, metabolic markers such as uric acid may also play a role in PCOS. Uric acid, the final product of purine metabolism, has pro-oxidant and anti-oxidant properties, depending on the microenvironment [11,12]. Moreover, it has a pro-inflammatory impact by inducing the NF-κB signaling pathway, which controls the transcription of many cytokines and pro-inflammatory molecules. Uric acid is also associated with insulin resistance and cardiovascular disease [13], conditions commonly comorbid with PCOS [14].

The present study aims to assess the predictive value of salivary testosterone, IL-6, IL-1β, TNF-α, and uric acid in distinguishing between girls with PCOS and healthy controls. By examining the relationships between these biomarkers and the hormonal and inflammatory profiles of PCOS, we seek to enhance the understanding of the underlying pathophysiology of this complex disorder and contribute to the development of non-invasive diagnostic tools. To our knowledge, this is the first study to evaluate multiple salivary biomarkers in adolescents with PCOS using ROC curve analysis.

Adolescents represent a unique group for whom the early diagnosis of PCOS is critical for timely interventions that can prevent the progression of long-term complications. However, invasive procedures such as blood draws may pose ethical and practical challenges in this population, potentially limiting participation in screening or research protocols. Our previous studies indicate that lifestyle modifications, particularly in diet and physical activity, can have a significant impact on managing PCOS symptoms and reducing the risk of these secondary conditions. By identifying PCOS early and implementing targeted lifestyle changes, patients may experience improvements in insulin sensitivity, hormonal balance, and inflammatory status, ultimately lowering the long-term burden of the disease [15,16,17,18,19]. These findings reinforce the value of non-invasive screening tools, such as salivary biomarkers, to facilitate early diagnosis and support effective preventive care strategies in PCOS management. Compared to serum-based testing, salivary diagnostics can be performed without venipuncture and may be more acceptable for repeated measurements in young populations.

## 2. Materials and Methods

All procedures performed in the study adhered to the principles outlined in the Declaration of Helsinki. The study was approved by the University Bioethics Committee (approval no. 536/18, dated 16 May 2018). Written, informed consent was obtained from all participants and, in the case of those under the age of 18, also from their legal guardians. All procedures were conducted in accordance with ethical standards, ensuring minimal discomfort and full confidentiality.

### 2.1. Study Population

Participants in the PCOS group were adolescents referred by outpatient gynecologists for inpatient evaluation due to clinically evident hyperandrogenism and irregular menstrual cycles. All were at least two years post-menarche and were newly diagnosed with PCOS during hospitalization. From August 2018 to September 2019, all adolescents hospitalized for diagnostic evaluation of suspected PCOS were consecutively screened. Those who met the inclusion criteria and provided consent were enrolled in the study. The inclusion criteria for the study group were based on NIH diagnostic guidelines, which require the presence of biochemical or clinical evidence of hyperandrogenism and ovulatory dysfunction, indicated by oligomenorrhea or amenorrhea. This approach is consistent with current recommendations for adolescents, which advise against relying on polycystic ovarian morphology due to its limited diagnostic value in this age group [2]. While ultrasound was performed, polycystic ovarian morphology was not a determining factor in diagnosis.

Exclusion criteria included the presence of any other systemic conditions, such as prolactin excess suggestive of pituitary adenoma, Cushing’s syndrome, thyroid disorders, congenital adrenal hyperplasia, androgen-secreting tumors, or use of hormonal therapy. Participants were also excluded if they used orthodontic appliances or antibacterial mouthwashes, were smokers, had poor oral hygiene, had complications from dental caries (e.g., signs of periapical inflammation), had severe gingivitis, or had taken antibiotics within the past six months. We aimed to recruit patients without sites of infection within the oral cavity to eliminate the potential influence of oral infections on salivary inflammatory markers.

Participants were excluded from the control group if they had any systemic diseases or presented symptoms suggestive of hormonal imbalance, such as amenorrhea, oligomenorrhea, irregular menstrual cycles, unusually heavy or short menstrual bleeding, moderate to severe acne (as assessed by the Investigator’s Global Assessment scale), or hirsutism (defined as a score > 7 on the modified Ferriman–Gallwey scale) [20,21]. Individuals who smoked, had poor oral hygiene, dental complications (e.g., periapical inflammation), severe gingivitis, used orthodontic appliances or antibacterial mouthwashes, or had been on antibiotics within the last six months were also excluded.

**Figure 1:**
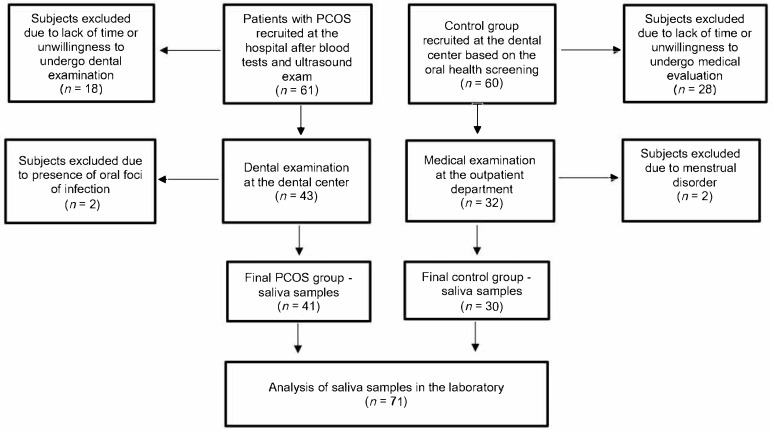
A flowchart of methodology.

The flowchart of the methodology is presented in Figure 1.

### 2.2. Medical and Dental Examination

Upon admission to the hospital, basic anthropometric measurements were taken for the study group. Excess body weight was assessed using BMI, according to the WHO growth reference standards for individuals aged 5–19 years [22]. Gynecological examinations, transabdominal ultrasonography, and blood sampling were conducted during the early follicular phase. Blood samples were drawn in the morning after an overnight fast. Laboratory analyses included measurements of luteinizing hormone (LH), follicle-stimulating hormone (FSH), estradiol, testosterone, dehydroepiandrosterone sulfate (DHEA-S), sex hormone-binding globulin (SHBG), cortisol, fasting glucose and insulin, as well as lipid profile components: triglycerides (TG), total cholesterol (TC), and high-density lipoprotein cholesterol (HDL-C).

The study was designed as a prospective observational investigation. Saliva samples were collected after the clinical diagnosis of PCOS had been established during hospitalization. Following a two-day hospital stay, patients were referred to the University Center of Stomatology and Specialist Medicine for oral health and hygiene assessment and for saliva collection.

The control group, consisting of healthy participants, was recruited from the same dental center following oral health screening. Those who met the inclusion criteria were referred for gynecological consultation at the hospital’s outpatient clinic. A gynecologist (G.J.B.) assessed them for clinical signs and symptoms of hyperandrogenism. Additionally, they were asked about the regularity of their menstrual cycles. A physical examination included measurements of height and weight, as well as a dermatological evaluation for hirsutism and acne. For ethical reasons, gynecological examinations and blood sampling were limited to the PCOS group to avoid subjecting healthy adolescents to unnecessary invasive procedures.

Oral hygiene was assessed by a calibrated pediatric dentist (N.W.), using the Silness–Löe plaque index (PI), while gingival health was evaluated using the Gingival Index (GI) on six teeth: 16, 12, 24, 36, 32, and 44. Oral hygiene scores were categorized as poor (PI = 1.9–3.0), average (PI = 0.7–1.8), or good (PI = 0–0.6). Gingival status was categorized as follows: 0—healthy; 0.1 to 1.0—mild gingivitis; 1.1 to 2.0—moderate gingivitis; and 2.1 to 3.0—severe gingivitis [23,24].

### 2.3. Saliva Assays

To minimize diurnal variation in testosterone and cytokine levels, saliva samples were collected during the morning hours (between 8.00 and 9:30 a.m.), with participants instructed to refrain from eating or drinking for at least two hours beforehand.

Unstimulated whole saliva was collected using the Salivette^®^ system (Sarstedt, Nümbrecht, Germany). Samples were centrifuged at 4000× *g* rpm for 10 min and then aliquoted and stored at −80 °C until further analysis. All samples were analyzed within 2 months of collection.

Due to the limited sample volume obtainable through unstimulated saliva collection, priority was given to the measurement of selected biomarkers. As a result, normalization using total protein or amylase was not performed.

### 2.4. Biochemical Parameters

Biochemical analyses of blood serum samples were performed at the hospital’s central laboratory. TC, HDL-C, and TG levels were measured with the use of an enzymatic colorimetric method (Roche Diagnostics GmbH, Mannheim, Germany). The level of low-density lipoprotein cholesterol (LDL-C) was estimated using the Friedewald formula: LDL-C (mg/dL) = TC − HDL-C − TG/5. Fasting glucose was measured with an enzymatic hexokinase method. Hormonal parameters, including insulin, LH, FSH, testosterone, 17-β-estradiol, DHEA-S, cortisol, and SHBG were measured using an electrochemiluminescence immunoassay method (ECLIA; Elecsys, Roche Diagnostics GmbH, Mannheim, Germany). The free androgen index (FAI) was calculated using total testosterone and SHBG levels using an online calculator (https://www.siemens-healthineers.com; accessed on 7 April 2020). Insulin resistance was estimated using the homeostasis model assessment (HOMA-IR), derived from fasting insulin and glucose concentrations [25]. All results were interpreted in the context of age- and sex-specific reference ranges and relevant literature [25,26,27,28]. There are no official reference values for the cortisol/DHEA-S ratio. However, in the study by Suh et al. [29], the mean ratio for women aged 19–29 was approximately 0.035, and in the study by Tomo et al. [30], healthy premenopausal women had DHEA-S levels about 15 times higher than cortisol, corresponding to a cortisol/DHEA-S ratio of 0.067. Based on these findings, we adopted the mean value of 0.05 as a reference point in our study of young women, dividing participants into two groups according to this cut-off.

A medical laboratory analyst (A.B.) from the Chair and Department of Medical Chemistry and Laboratory Medicine at Poznań University of Medical Sciences carried out all analyses of inflammatory biomarkers and salivary testosterone concentrations. Salivary IL-6, IL-1β, and TNF-α were quantified using commercially available ELISA kits (Shanghai Sunred Biological Technology Co., Shanghai, China). Salivary testosterone levels were measured using an ELISA kit from DRG International Inc. (Springfield, NJ, USA), with absorbance readings performed on a TECAN-SUNRISE reader with Magellan software. The manufacturer-reported reference range for salivary testosterone in adult females aged 21–30 is 7.9–50.4 pg/mL (5th–95th percentile), with a median of 20.8 pg/mL based on measurements in 40 apparently healthy women. Reference data for females under 21 years of age are not available.

The uric acid concentration was evaluated via enzymatic colorimetric assays using a COBAS Integra 400 Plus analyzer (Roche Diagnostics).

The precision of the applied assays, expressed as intra- and inter-assay coefficients of variation (CV), was below 10% for salivary testosterone and cytokines, and below 5% for uric acid, respectively.

### 2.5. Statistical Analysis

Data were analyzed using MedCalc^®^ Statistical Software version 20.115 (MedCalc Software Ltd., Ostend, Belgium; https://www.medcalc.org; 2022; accessed on 5 November 2024), with statistical significance set to *p* < 0.05. No formal adjustment for multiple comparisons was applied, given the exploratory nature of the study.

The calculation of sample size required for the ROC curve analysis was based on the following assumptions: the area under the curve (AUC) for the null hypothesis was set to 0.50 (indicating no discrimination), while the expected AUC was anticipated to be 0.75, reflecting a moderate diagnostic performance. A significance level (α) of 0.05 and a statistical power (1-β) of 0.80 were used to ensure adequate sensitivity in detecting a meaningful difference. The ratio of sample sizes between the negative (control) and positive (PCOS) groups was set to 1:1, assuming equal group sizes. Based on these data, the minimum required sample size was calculated to be 19 participants in the positive (PCOS) group and 19 participants in the negative (control) group to detect a statistically significant difference in AUC with the specified power and significance level.

Seventy-one participants were recruited for the study, consisting of 41 girls with PCOS and 30 healthy controls. While we aimed for equal sample sizes, the recruitment of healthy controls proved challenging, mainly due to difficulties in motivating healthy girls to visit the gynecological hospital’s outpatient clinic for the study.

The statistical analysis included the Mann–Whitney U test and *t*-tests to compare group variables. The Shapiro–Wilk test was conducted to confirm the normality of data distribution. Variables with a normal distribution were presented as mean ± standard deviation (SD), while non-normally distributed variables were reported as median and interquartile range (Q1; Q3). The Mann–Whitney U test was used for continuous data that did not follow a normal distribution. In cases where data were normally distributed, an independent samples *t*-test was applied. To evaluate the relationship between salivary and serum testosterone levels, Pearson’s correlation coefficient was applied, as both variables demonstrated normal distribution.

ROC curve analysis was performed to evaluate the diagnostic performance of salivary biomarkers, including testosterone, cytokines, and uric acid, in distinguishing between girls with PCOS and healthy controls. Additionally, ROC curve analyses were used to assess the diagnostic utility of salivary biomarkers in predicting some hormonal and metabolic abnormalities in the PCOS group, selected based on the initial comparisons of subgroups sharing similar hormonal and metabolic characteristics. The area under the curve (AUC) was calculated for each biomarker, and the optimal cut-off points were determined by maximizing Youden’s index to balance sensitivity and specificity. AUC values were reported to assess the diagnostic accuracy of each biomarker.

## 3. Results

The study group included 41 adolescent females between the ages of 15 and 19, recruited by pediatric and adolescent gynecology specialists from the Gynecology and Obstetrics Hospital at Poznan University of Medical Sciences (Greater Poland Voivodeship, Poland). The control group consisted of 30 healthy females aged 15 to 19, recruited from the University Center of Stomatology and Specialist Medicine in Poznan, following oral health screening. The control participants were matched with the PCOS group based on age and oral hygiene.

### 3.1. Clinical and Hormonal Characteristics of PCOS Participants

Among participants with PCOS, 6 (14.6%) exhibited polycystic ovarian morphology on ultrasound. All 41 (100%) met the NIH diagnostic criteria, presenting with oligomenorrhea and clinical hyperandrogenism. Hirsutism was classified as mild in 32 participants (78.1%), moderate in 8 (19.5%), and severe in 1 (2.4%). Acne severity was reported as severe in 18 participants (43.9%), moderate in 22 (53.7%), and mild in 1 (2.4%) [19,20].

### 3.2. Comparison of Salivary Biomarkers and Clinical Parameters Between PCOS and Control Groups

Table 1 presents subjects’ characteristics, including age, BMI, salivary biomarker concentrations, PI, and GI.

In the study group, twenty-two subjects were classified as overweight, while only five subjects in the control group had a BMI above one standard deviation of the growth reference median. PCOS participants had a significantly higher BMI (25.2 vs. 19.9 kg/m^2^, *p* < 0.0001) and elevated levels of salivary biomarkers, including testosterone (41.81 vs. 28.74 pg/mL, *p* < 0.0001), TNF-α (16.69 vs. 12.04 pg/mL, *p* < 0.0001), IL-6 (9.46 vs. 5.93 pg/mL, *p* < 0.0001), and IL-1β (170.82 vs. 126.46 pg/mL, *p* < 0.0001). PCOS participants had significantly lower uric acid levels (137.50 vs. 217.24 µmol/L, *p* = 0.0001).

Oral health indices (GI and PLI) showed no significant differences between groups. Most patients had GI < 1, and only five patients had moderate gingivitis (GI > 1): three in the study group and two in the control group.

### 3.3. Serum Hormonal and Metabolic Profiles in PCOS

Table 2 summarizes the serum hormonal and biochemical parameters of the PCOS group.

Among the PCOS subjects, mean FSH levels were 5.08 ± 2.03 mIU/mL, with 11 participants having values below the reference range of 3.5–12.5 mIU/mL. LH levels had a median of 10.97 mIU/mL (6.55; 18.06), where three subjects were below and 19 above the normal range (2.4–12.6 mIU/mL). The LH/FSH ratio showed a median of 2.29 (1.27; 3.35), with 23 individuals exceeding the reference value of ≤2.

For estradiol, the median was 46.70 pg/mL (36.89; 70.16), with five individuals showing elevated levels compared to the follicular phase reference range (12.5–166 pg/mL). Testosterone was elevated in 21 subjects, with a mean of 53.76 ± 19.40 ng/dL, exceeding the reference value of 51 ng/dL. SHBG had a median of 41.11 nmol/L (24.85; 56.54), with 11 subjects falling below the reference range (26.1–110 nmol/L).

FAI showed a median of 4.10 (2.97; 10.05), with 19 subjects presenting androgen excess. DHEA-S levels averaged 7.37 ± 3.03 µmol/L, with 10 subjects above the reference limit (1.77–9.99 µmol/L). Cortisol had a mean of 411.65 ± 122.97 nmol/L, with nine individuals above and two below the reference range (166–507 nmol/L). The cortisol/DHEA-S ratio showed a median of 0.06 (0.04; 0.08), with 21 subjects showing a value above 0.05.

In metabolic parameters, fasting glucose levels averaged 88.65 ± 5.98 mg/dL, all within the reference range (60–99 mg/dL). Fasting insulin levels were elevated in seven individuals, with a mean of 14.96 ± 11.32 mU/L (reference: 2.6–24.9 mU/L), and HOMA-IR index of insulin resistance in 33 subjects with a median value of 3.35 (2.48; 4.38) (reference < 2.32).

Lipid profiles showed average TC levels of 158.2 ± 24.6 mg/dL, with four subjects above the reference of <190 mg/dL. HDL-C averaged 53.1 ± 8.3 mg/dL, with five subjects below the ≥45 mg/dL threshold. LDL-C averaged 85.8 ± 20.5 mg/dL, with four subjects exceeding the recommended < 115 mg/dL level. TG had a median of 87.8 mg/dL (69.78; 118.5), with six subjects above the reference value of <150 mg/dL.

A significant positive correlation was observed between salivary and serum testosterone levels (Pearson’s r = 0.4664, *p* = 0.0021).

### 3.4. Diagnostic Performance of Salivary Biomarkers in PCOS

Table 3 displays an ROC analysis of salivary biomarkers in PCOS.

In the group of all subjects, salivary TNF-α showed the highest diagnostic accuracy with an AUC of 0.921 (*p* < 0.0001), 90.24% sensitivity, and 86.67% specificity, with a Youden index of 0.769 at a cut-off of >13.8 pg/mL. IL-6 also had high diagnostic accuracy, with an AUC of 0.891 (*p* < 0.0001), 87.80% sensitivity, 73.33% specificity, and a Youden index of 0.611 at a cut-off > 6.8 pg/mL. IL-1β had an AUC of 0.870 (*p* < 0.0001), achieving approximately 83% for sensitivity and specificity, and a Youden index of 0.663 at >147.1 pg/mL. Salivary testosterone had an AUC of 0.797 (*p* < 0.0001), showing high specificity (96.67%) but lower sensitivity (53.66%) at a cut-off > 38.3 pg/mL. Uric acid showed moderate diagnostic value with an AUC of 0.773 (*p* < 0.0001), 60.98% sensitivity, 86.67% specificity, and a Youden index of 0.476 at a cut-off ≤ 152 µmol/L.

In the normal-weight subgroup, TNF-α had an AUC of 0.891 (*p* < 0.0001), sensitivity of 89.49%, specificity of 80%, and a Youden index of 0.695 at >13.6 pg/mL. IL-1β also showed an AUC of 0.880 (*p* < 0.0001), sensitivity of 89.47%, specificity of 88%, and a Youden index of 0.775 at >149.9 pg/mL. IL-6 showed an AUC of 0.896 (*p* < 0.0001), with 63.16% sensitivity, 100% specificity, and a Youden index of 0.632 at a cut-off of >8.8 pg/mL. Salivary testosterone showed an AUC of 0.806 (*p* < 0.0001), with 47.39% sensitivity and 100% specificity at a cut-off > 38.3 pg/mL. Uric acid presented an AUC of 0.766 (*p* = 0.0002), sensitivity of 52.63%, specificity of 92%, and a Youden index of 0.446 at ≤149.6 µmol/L.

### 3.5. Associations Between Salivary Biomarkers and Clinical or Hormonal Variables in PCOS

Table 4 compares salivary testosterone, TNF-α, IL-1β, IL-6, and uric acid levels across various clinical characteristics, such as BMI, testosterone levels, free androgen index (FAI), estradiol, LH/FSH ratio, HOMA-IR, and lipidogram status (all fractions within reference values vs. at least one fraction with abnormal values).

No significant differences were observed between normal and overweight groups across all markers, although TNF-α levels showed a trend (*p* = 0.0798) toward higher values in the overweight group.

Elevated testosterone in the blood was associated with slightly higher salivary testosterone and TNF-α, though not statistically significant. Other markers showed no significant differences (*p* > 0.05).

A significant increase in TNF-α (*p* = 0.0121) and IL-6 (*p* = 0.0214) was found in the group with elevated FAI. Salivary testosterone levels were significantly higher in individuals with elevated estradiol (*p* = 0.0257), while other markers did not show significant differences.

Individuals with a high LH/FSH ratio had significantly elevated salivary testosterone (*p* = 0.0292), but no substantial changes in other markers.

No significant differences in inflammatory or biochemical markers were found between groups with normal and elevated HOMA-IR or different lipidogram status.

### 3.6. Predictive Utility of Salivary Biomarkers for Hormonal and Metabolic Abnormalities in PCOS

Table 5 presents the results of the ROC analysis for predicting hormonal and metabolic abnormalities in PCOS patients using various salivary biomarkers.

Salivary testosterone showed a significant predictive value for an abnormal LH/FSH ratio, with an AUC of 0.700 (*p* = 0.0234), achieving 73.91% sensitivity and 72.22% specificity at a cut-off level of >37.2 pg/mL. In contrast, other markers, including TNF-α, IL-1β, IL-6, and uric acid, displayed lower predictive capacities for this ratio, with AUC values below 0.64, all of which were not statistically significant.

Salivary testosterone showed significant predictive value for abnormal LH/FSH ratios in the normal-weight PCOS subgroup, while IL-6 had an AUC of 0.733, approaching statistical significance (*p =* 0.0614).

Only TNF-α and IL-6 demonstrated statistically significant predictive capability for predicting an elevated FAI, with TNF-α yielding an AUC of 0.730 (*p* = 0.0042) and IL-6 an AUC of 0.711 (*p* = 0.0104). Salivary testosterone had a moderate AUC of 0.658, approaching statistical significance (*p* = 0.0749).

For the entire PCOS cohort, uric acid demonstrated the highest predictive accuracy for a high cortisol/DHEA-s ratio, with an AUC of 0.698 (*p* = 0.0223), sensitivity of 57.14%, and specificity of 85% at a cut-off of >156.78 µmol/L. Other biomarkers showed lower non-significant predictive values. In the normal-weight PCOS subgroup, uric acid again emerged as the strongest predictor, with an AUC of 0.828 (*p* = 0.0030), achieving sensitivity and specificity values of 80% and 88.89%, respectively, at a cut-off of >149.57 µmol/L.

Salivary testosterone proved effective for predicting elevated estrogen levels, achieving a statistically significant AUC of 0.811 (*p* < 0.0001) with 100% sensitivity and 69.44% specificity at a> 44.40 pg/mL threshold. Other biomarkers, including TNF-α, IL-1β, and IL-6, had lower AUC values and no statistical significance

## 4. Discussion

Many studies indicate a close link between chronic low-grade inflammation and the development of PCOS [31]. Pro-inflammatory cytokines, including tumor necrosis factor-alpha (TNF-α), interleukin-6 (IL-6), and interleukin-1 (IL-1β), are key inflammatory mediators and are frequently found at elevated levels in subsets of women with PCOS [10,32,33,34,35,36]. TNF-α, produced by the corpus luteum, exhibits varying levels throughout the menstrual cycle [37]. These cytokines are believed to play essential roles in reproductive functions, such as ovarian steroidogenesis, follicle maturation, and ovulation—all of which are commonly disrupted in PCOS [31].

PCOS and insulin resistance are often linked to obesity, with adipose tissue being a key source of pro-inflammatory mediators, which can drive chronic inflammation. Subcutaneous adipose tissue has an elevated androgen production rate, potentially contributing to the hyperandrogenism associated with PCOS [31]. Additionally, inflammation within adipose tissue itself has been connected to PCOS-related hyperandrogenism. Deligeoroglou et al. [38] suggest that androgen-induced adipocyte hypertrophy may narrow stromal arteries, creating tissue hypoxia that then triggers inflammation.

While numerous studies report elevated inflammatory markers in women with PCOS, it remains uncertain whether these increases are directly associated with PCOS or are influenced by factors like obesity and abdominal fat distribution. In the meta-analysis published by Escobar-Morreale et al., the degree of elevation in circulating levels of CRP and IL-6 in PCOS was much higher when obesity was also present [39], and some studies showed that serum CRP elevations in women with PCOS were no longer statistically significant when controlling for body mass index [40], or that obesity alone is responsible for the systemic low-grade inflammation [41]. Studies by Gonzalez et al. and Vasyukova et al. indicate the presence of inflammation in normal-weight women with PCOS [33,42].

Persistent inflammation in PCOS, observed in both overweight and normal-weight patients, may have genetic underpinnings. Research has linked specific pro-inflammatory genotypes, such as those associated with TNF-α, and IL-6, to the syndrome [32,43].

Regardless of the pathogenesis, this ongoing low-grade inflammation interacts with hyperandrogenism, obesity, and insulin resistance in PCOS, contributing to metabolic complications and elevating the risk for type 2 diabetes and cardiovascular disease [4].

Improving diagnostic methods is crucial because it helps to implement treatment early when complications associated with the condition can be prevented. To date, research on salivary biomarkers in PCOS has been limited. The only prior exploratory report, a non–peer-reviewed conference abstract by Cooper et al. [44], assessed salivary testosterone, cortisol, DHEA-S, and CRP in adult women with and without PCOS, identifying elevated testosterone and CRP in PCOS alongside associations with BMI, ethnicity, and oral contraceptive use. This work supports the feasibility of saliva as a diagnostic medium for endocrine and inflammatory profiling in PCOS. Our findings contribute novel, peer-reviewed evidence in an adolescent population by assessing the diagnostic potential of salivary testosterone, TNF-α, IL-1β, IL-6, and uric acid in both the overall cohort and a subset of normal-weight participants. Our ROC analysis revealed that salivary TNF-α, IL-6, and IL-1β demonstrated high sensitivity and specificity for PCOS detection, confirming the significance of inflammatory markers in PCOS pathophysiology. Notably, their strong diagnostic performance in normal-weight individuals suggests that inflammation in PCOS is present even in the absence of overweight or obesity. As shown in Table 3, the cut-off values for salivary biomarkers in this subgroup were similar to those in the full cohort, with no clinically relevant differences. Salivary testosterone also emerged as a significant marker, characterized by high specificity but lower sensitivity, indicating its utility in confirming hyperandrogenism—a hallmark feature of PCOS. Importantly, the observed correlation between salivary and serum testosterone reinforces the validity of salivary assays as a reflection of systemic hyperandrogenism. Our salivary testosterone values are similar to those reported by Youssef et al., who also used the DRG ELISA assay and found significantly elevated levels in adult women with PCOS compared to controls (41.45 ± 19.43 pg/mL vs. 16.15 ± 6.56 pg/mL, *p* < 0.0001) [45].

An unexpected finding of this study was the observation of lower salivary uric acid levels in adolescents with PCOS. This contrasts with several serum-based studies in adult women, where higher uric acid concentrations were reported in PCOS populations, as noted by Quiñónez et al., Mu et al., and Yarali et al. [46,47,48]. Conversely, other studies, including those by Anttila et al. and Luque-Ramirez et al., found no significant differences in serum uric acid levels between PCOS and control groups [49,50]. Notably, one study reported that a subgroup of women with PCOS characterized by hyperandrogenism and anovulation exhibited lower plasma uric acid levels compared to healthy controls [51], which is consistent with our findings.

These discrepancies may, in part, reflect age-related physiological differences, as uric acid metabolism can differ between adolescents and adults [12]. Moreover, dietary patterns, which were not assessed in this study, could have influenced uric acid levels. Recent research indicates that uric acid concentrations are increased not only by purine-rich diets but also by excessive fructose consumption, a common feature of adolescent diets [52]. On the other hand, diets lower in animal protein, such as vegetarian diets, have been associated with reduced uric acid concentrations, as shown by Jakse et al. [53]. Furthermore, Yuan et al. found that higher physical activity levels were linked to lower uric acid concentrations [54]. These lifestyle factors may be relevant in adolescents with PCOS, among whom dietary choices aimed at improving metabolic health or controlling weight are common [19]. However, we did not assess diet, hydration, or physical activity, which may affect salivary uric acid levels and limit interpretation [14,53].

It must be remembered that hormonal factors, including elevated estrogen levels in PCOS, may also influence uric acid metabolism, potentially reducing its levels in affected individuals. Estrogen is known to reduce uric acid levels by enhancing renal excretion [14]. While we did not observe differences in uric acid levels between subjects with normal and elevated estradiol levels, the long-term hormonal profile of the study population and its potential influence on the assessed parameters remain unknown.

Given the current evidence, the clinical utility of salivary uric acid as a biomarker for PCOS remains uncertain. Further studies are needed to clarify its role, considering dietary habits, hormonal status, age-related metabolic factors, and the physiological differences between salivary and serum uric acid dynamics.

On the other hand, recent evidence supports the diagnostic relevance of redox-related salivary biomarkers in PCOS. Gholizadeh et al. [55] reported significantly reduced levels of salivary superoxide dismutase in women with PCOS, suggesting impaired antioxidant defense mechanisms detectable in saliva. In line with this, our finding of reduced salivary uric acid in the PCOS group may reflect altered oxidative stress balance, given that uric acid acts as a major non-enzymatic antioxidant in saliva.

Interestingly, salivary uric acid levels were found to be associated with the cortisol/DHEA-S ratio in the blood of girls with PCOS, suggesting potential interactions between metabolic and stress-related responses in this condition. Studies show that an elevated cortisol-to-DHEA-S ratio is linked to an increased risk of metabolic syndrome, where high cortisol levels are associated with metabolic dysfunctions, while DHEA-S appears to play a protective role [56]. A high cortisol-to-DHEA-S ratio has also been associated with stress and immune dysregulation, further highlighting the impact of this hormonal balance on overall health [57,58,59].

Some studies propose that higher adrenal androgen levels, such as DHEA-S, contribute to a more favorable metabolic profile, which may mitigate some PCOS-related risks [60]. Elevated levels of DHEA-S are linked to improved lipid profiles and insulin sensitivity in some PCOS populations, possibly due to its anti-inflammatory and metabolic regulatory roles. Increased DHEA-S levels are observed in approximately 11% of females with PCOS presenting hyperandrogenism and only 2.6% of females with phenotype D without hyperandrogenism (polycystic ovaries + oligo-anovulation) [61]. In our study, as many as 24.4% of females had an increased level of DHEA-s in the blood, and all represented phenotypes with hyperandrogenism (5 phenotype A: polycystic ovaries + hyperandrogenism + oligo-anovulation, 36 phenotype B: hyperandrogenism + oligo-anovulation).

In parallel, uric acid levels have been associated with metabolic irregularities. For example, Lucas et al. found that salivary uric acid levels rose significantly in individuals experiencing acute social stress, suggesting that uric acid may contribute to the body’s response to psychological stress [62].

The positive correlation between uric acid and the cortisol/DHEA-S ratio suggests that girls with PCOS who have a higher cortisol-to-DHEA-S ratio also tend to have elevated uric acid levels. This correlation may imply that uric acid could be linked to androgenic activity and adrenal function in PCOS or that both increased uric acid levels and a high cortisol/DHEA-S ratio share similar underlying mechanisms.

One may wonder whether the relatively high DHEA-S concentrations in our study are due to the young age of the patients or whether they are related to the favorable metabolic profile of the study group, which manifested in a low salivary uric acid level compared to the control group. Nevertheless, the observed high specificity of uric acid in predicting a high cortisol/DHEA-S ratio, especially in normal-weight PCOS individuals, suggests its potential utility in identifying patients with hormonal patterns often linked to metabolic syndrome, immune dysfunction, and certain psychiatric disorders [56,57,58,59].

Our findings suggest that salivary TNF-α, IL-6, and IL-1β offer robust diagnostic value, while salivary testosterone and uric acid contribute additional specificity to a multi-marker diagnostic approach. This panel of salivary biomarkers represents a promising, non-invasive tool for the early detection and deeper understanding of PCOS pathophysiology, potentially supporting stratified approaches to diagnosis and management, particularly in younger populations or those who prefer non-invasive testing methods.

The early diagnosis of endocrinopathies is essential for timely intervention and improved patient outcomes. Unlike other conditions, such as growth hormone deficiency, which necessitates multiple blood tests [63], PCOS presents a unique opportunity for non-invasive screening conducted through salivary analysis.

However, certain limitations should be considered when interpreting these results. The cross-sectional design restricts conclusions regarding causality and temporal changes in biomarker levels. The relatively small sample size and the absence of blood tests in healthy controls—who did not have a clinical indication for venipuncture—may limit generalizability and prevent direct systemic comparisons. This also means that we cannot determine whether salivary biomarker levels in healthy individuals have the same systemic relevance as observed in the PCOS group. Future studies should include systemic biomarker assessments in control groups, either through ethically approved blood sampling in young adults or by using residual samples from routine clinical care, to enable direct comparisons between salivary and serum markers. Furthermore, the predominance of “classic” PCOS phenotypes (A and B) in our cohort may reduce applicability to milder phenotypes lacking hyperandrogenism or ovulatory dysfunction, such as phenotype C (ovulatory hyperandrogenism) or phenotype D (non-hyperandrogenic). In addition, the relatively small number of participants in certain subgroup analyses (e.g., elevated FAI and cortisol/DHEA-S ratio) may have limited statistical power and increased the risk of type II errors when detecting more subtle biomarker associations. Unmeasured factors such as dietary habits, psychological stress, and long-term hormonal variability could also have influenced salivary biomarker levels. Although we explored estradiol’s role in uric acid metabolism, limited cases with elevated estradiol prevented more detailed analyses.

Another limitation is the lack of Anti-Müllerian Hormone (AMH) assessment, a promising serum biomarker for PCOS. A recent review by Vale-Fernandes et al. [64] emphasizes its diagnostic value alongside methodological challenges, including assay variability and population-specific cut-offs.

The cut-off values derived from our ROC analysis demonstrated promising diagnostic potential for distinguishing girls with PCOS from healthy controls. However, these thresholds were established using laboratory-based ELISA assays and require further validation in larger populations. Their clinical application will also depend on the future development of standardized, point-of-care salivary tests.

To establish salivary assays as a reliable diagnostic tool for PCOS, future research should focus on larger, multicenter, longitudinal studies encompassing diverse age groups and PCOS phenotypes. Direct comparisons between salivary and serum biomarkers, alongside evaluations of diagnostic accuracy, cost-effectiveness, and clinical utility, are essential. Additionally, incorporating lifestyle and stress-related variables will further clarify the role of salivary biomarkers in diagnosis and potential disease monitoring.

In recent years, there has been growing interest in identifying novel biomarkers for PCOS. Examples include elevated serum calprotectin levels [65] and oxidative stress detectable even in normal-weight patients [66]. As highlighted by Singh et al., combining different types of biomarkers may improve diagnostic precision and support more personalized approaches to PCOS [67]. Our study reflects this ongoing direction in PCOS research.

## 5. Conclusions

In conclusion, this preliminary study highlights the potential of a salivary biomarker panel as a supportive, non-invasive screening tool for PCOS in adolescents, with diagnostic utility confirmed both in the general population and in normal-weight girls. These findings lay the groundwork for future research aimed at validating salivary assays as part of early detection strategies and personalized care in PCOS management across different nutritional statuses.

## Figures and Tables

**Table 1:** Characteristics of the PCOS group and control subjects.

Characteristic	PCOS (*n* = 41)	Controls (*n* = 30)	*p*-Value
Age (years)	16 (16; 17)	16 (15; 17)	0.2031
BMI (kg/m^2^)	25.2 (21.2; 30.1)	19.9 (18.0; 22.0)	<0.0001
Salivary biomarkers			
Testosterone (pg/mL)	41.81 ± 12.20	28.74 ± 7.59	<0.0001
TNF-α (pg/mL)	16.69 (14.83; 19.03)	12.04 (10.79; 13.22)	<0.0001
IL-6 (pg/mL)	9.46 (7.18; 12.85)	5.93 ± 1.66	<0.0001
IL-1β (pg/mL)	170.82 (151.13; 194.13)	126.46 (106.10; 143.02)	<0.0001
Uric acid (µmol/L)	137.50 (120.19; 193.40)	217.24 (159.20; 304.07)	0.0001
Oral health indices			
GI	0.33 (0.13; 0.72)	0.25 (0.08; 0.58)	0.3214
PI	0.63 (0.25; 0.89)	0.71 (0.17; 1.04)	0.9582

Data are presented as means ± SDs or medians (Q1; Q3), *p*-values based on *t*-test or Mann–Whitney U test, BMI—body mass index, TNF-α- tumor necrosis factor α, IL-6—interleukin 6, IL-1β—interleukin 1β, GI—gingival index, PI—plaque index.

**Table 2:** Biochemical and hormonal profile of PCOS participants.

Parameter	Mean ± SD or Median (Q1; Q3)	Reference Values	Abnormal Values [*n*]
FSH (mIU/mL)	5.08 ± 2.03	3.5–12.5 *	11 ↓
LH (mIU/mL)	10.97 (6.55; 18.06)	2.4–12.6 *	3 ↓ 19 ↑
LH/FSH	2.29 (1.27; 3.35)	≤2 [27]	23 ↑
estradiol (pg/mL)	46.70 (36.89; 70.16)	12.5–166 * (follicular phase)	5 ↑
testosterone (ng/dL)	53.76 ± 19.40	<51 [28]	21 ↑
SHBG (nmol/L)	41.11 (24.85; 56.54)	26.1–110 *	11 ↓
FAI	4.10 (2.97; 10.05)	≤4.4 [26]	19 ↑
DHEA-S (µmol/L)	7.37 ± 3.03	1.77–9.99 *	10 ↑
cortisol (nmol/L)	411.65 ± 122.97	166–507 *	2 ↓ 9 ↑
cortisol/DHEA-S ratio	0.06 (0.04; 0.08)	<0.05 [29,30]	21 ↑
fasting glucose (mg/dL)	88.65 ± 5.98	60–99 *	0 ↑
fasting insulin (mU/L)	14.96 ± 11.32	2.6–24.9 *	7 ↑
HOMA-IR	3.35 (2.48; 4.38)	<2.32 [25]	33 ↑
TC (mg/dL)	158.2 ± 24.6	<190 *	4 ↑
HDL-C (mg/dL)	53.1 ± 8.3	≥45 *	↓ 5
LDL-C (mg/dL)	85.8 ± 20.5	<115 *	4 ↑
TG (mg/dL)	87.8 (69.78; 118.5)	<150 *	6 ↑

FSH—follicle-stimulating hormone, LH—luteinizing hormone, FAI—free androgen index, SHBG—sex hormone-binding globulin, DHEA-S—dehydroepiandrosterone sulfate, HOMA-IR—Homeostatic Model Assessment of Insulin Resistance, TC—total cholesterol, HDL-C—high-density lipoprotein cholesterol, LDL-C—low-density lipoprotein cholesterol, TG—triglycerides, * according to the hospital age- and sex-specific laboratory reference ranges, ↑ above, ↓ below the reference values.

**Table 3:** ROC results for salivary biomarkers in PCOS in all and normal-weight participants.

	AUC	*p*-Value	Sensitivity	Specificity	Youden Index	Associate Criterion (Cut-Off)
All subjects						
Salivary testosterone	0.797	<0.0001	53.66	96.67	0.502	>38.3 pg/mL
TNF-α	0.921	<0.0001	90.24	86.67	0.769	>13.8 pg/mL
IL-1β	0.870	<0.0001	82.93	83.33	0.663	>147.1 pg/mL
IL-6	0.891	<0.0001	87.80	73.33	0.611	>6.8 pg/mL
Uric acid	0.773	<0.0001	60.98	86.67	0.476	≤152 µmol/L
Subjects with normal weight						
Salivary testosterone	0.806	<0.0001	47.39	100.00	0.474	>38.3 pg/mL
TNF-α	0.891	<0.0001	89.49	80.00	0.695	>13.6 pg/mL
IL-1β	0.880	<0.0001	89.47	88.00	0.775	>149.9 pg/mL
IL-6	0.896	<0.0001	63.16	100.00	0.632	>8.8 pg/mL
Uric acid	0.766	0.0002	52.63	92.00	0.446	≤149.6 µmol/L

ROC—receiver operating characteristic, AUC—area under the curve, TNF-α- tumor necrosis factor α, IL-6- interleukin 6, IL-1β—interleukin 1β.

**Table 4:** Comparison of salivary biomarkers across different characteristics.

Characteristic	Salivary Testosterone (pg/mL)	TNF-α (pg/mL)	IL-1β (pg/mL)	IL-6 (pg/mL)	Uric Acid (µmol/L)
BMI					
normal *n* = 19	42.19± 13.99	15.90 (14.78; 18.34)	170.82 (160.66; 184.63)	9.46 (7.53; 11.12)	149.57 (127.93; 204.55)
overweight *n* = 22	41.48 ± 10.76	17.91 (15.96; 20.60)	171.06 (148.20; 194.13)	9.17 (7.17; 16.33)	136.30 (113.63; 162.22)
*p*-value	0.8523	0.0798	0.7437	0.9791	0.3740
Serum testosterone					
<51 ng/dL *n* = 20	35.46 (30.69; 44.87)	16.52 (14.87; 19.01)	179.33 ± 64.76	8.44 (7.02; 11.81)	152.51 ± 52.21
≥51 ng/dL *n* = 21	45.35 (35.29; 54.55)	17.23 (14.83; 19.45)	174.20± 20.26	9.59 (8.16; 15.01)	149.57 (112.43; 208.42)
*p*-value	0.1552	0.5060	0.7306	0.3090	0.8449
FAI					
≤4.4 *n* = 22	35.46 (28.75;45.35)	15.80 (14.50; 17.27)	183.78 ± 60.10	8.29 (6.99; 10.92)	140.64 (122.18; 206.35)
>4.4 *n* = 19	45.28 ± 11.64	18.69 (16.18; 23.74)	168.50 ± 23.47	9.79 (8.63; 17.82)	137.50 (116.92; 167.54)
*p*-value	0.0844	**0.0121**	0.3049	**0.0214**	0.6853
Estradiol					
≤166 pg/mL *n* = 36	37.06 (30.69; 49.35)	16.60 (14.67; 19.04)	170.82 (150.16; 194.35)	8.94 (7.18; 11.96)	137.50 (113.93; 195.31)
>166 pg/mL *n* = 5	52.39 ± 6.91	17.23 (15.91; 27.91)	180.91 (146.90; 185.97)	13.32 ± 6.59	152.20 (136.28; 176.65)
*p*-value	**0.0257**	0.4733	0.9682	0.4141	0.4856
LH/FSH ratio					
≤2 *n* = 18	33.77 (27.62; 44.40)	16.52 (15.71; 18.87)	182.78 (152.11; 215.88)	8.78 (7.19; 10.92)	165.90 (126.23; 224.72)
>2 *n* = 23	45.30 ± 11.36	17.23 (14.78; 20.22)	170.32 (150.53; 18.23)	9.60 (7.45; 18.19)	136.30 (110.19; 155.64)
*p*-value	**0.0292**	0.5198	0.1411	0.3181	0.1309
Cortisol					
≤507 nmol/L *n* = 32	41.76 ± 11.96	16.82 (15.39; 19.84)	170.82 (150.16; 189.61)	8.82 (7.11; 11.34)	137.50 (118.20; 202.75)
>507 nmol/L *n* = 9	41.99 ± 13.80	16.09 ± 2.39	180.91 (160.65; 199.56)	12.91± 5.91	151.64 ± 48.97
*p*-value	0.9604	0.2252	0.5600	0.2134	1.0000
DHEA-S					
≤9.99 µmol/L *n* = 31	40.50 ± 12.12	16.51 (14.88; 19.05)	170.32 (148.20; 184.63)	8.97 (7.22; 13.36)	152.20 (117.17; 220.13)
>9.99 µmol/L *n* = 10	45.86 ± 12.18	18.78 (14.86; 19.01)	177.30 (169.83; 201.13)	9.69 (7.05; 12.43)	126.87 (122.18; 13.75)
*p*-value	0.2319	0.6058	0.1670	0.6488	0.0980
cortisol/DHEA-S ratio					
<0.05 *n* = 20	42.84 ± 12.56	17.64 (15.06; 19.04)	184.82 ± 50.78	8.82 (70.20; 11.2)	128.35 (111.24; 146.68)
>0.05 *n* = 21	40.83 ± 12.08	16.52 (14.69; 19.22)	167.84 (148.20; 182.90)	9.58 (7.43; 17.89)	181.17 ± 76.58
*p*-value	0.6039	0.6294	0.1589	0.3411	**0.0304**
HOMA-IR					
<2.32 *n* = 8	42.25 (29.93; 54.08)	15.62 (14.81; 17.60)	160.42 ± 47.13	8.55 (7.16; 11.77)	143.54 (96.76; 228.34)
>2.32 *n* = 33	38.65 (32.18; 51.67)	16.96 (15.09; 19.27)	171.80 (156.17; 194.24)	9.59 (7.18; 13.41)	137.50 (124.27; 181.69)
*p*-value	0.8693	0.4015	0.3483	0.5106	0.9869
Lipidogram					
normal *n* = 31	41.03 (32.77; 53.51)	16.51 (14.63; 19.01)	170.82 (157.92; 183.25)	8.97 (7.08; 11.35)	143.78 (122.88; 188.22)
abnormal *n* = 10	37.91 (30.83; 45.35)	16.90 (16.14; 19.07)	177.71 (148.20; 222.42)	10.38 (8.59; 18.16)	131.87 (114.23; 206.35)
*p*-value	0.6164	0.4299	0.5138	0.1917	0.6819

Data are presented as means ± SDs or medians (Q1; Q3), *p*-values based on *t*-test or Mann–Whitney U test (statistically significant differences in bold), BMI—body mass index, TNF-α—tumor necrosis factor α, IL-6—interleukin 6, IL-1β—interleukin 1β, FAI—free androgen index, LH—luteinizing hormone, FSH—follicle-stimulating hormone, DHEA-S—dehydroepiandrosterone sulfate, HOMA-IR—homeostatic model assessment of insulin resistance.

**Table 5:** Results of the ROC analysis for salivary biomarkers in predicting hormonal and metabolic abnormalities in PCOS.

	AUC	*p*-Value	Sensitivity	Specificity	Youden Index	Associate Criterion (Cut-Off)
All PCOS subjects—prediction of abnormal LH/FSH ratio						
Salivary testosterone	0.700	**0.0234**	73.91	72.22	0.461	>37.2 pg/mL
TNF-α	0.559	0.5278	39.13	83.33	0.225	>18.9 pg/mL
IL-1β	0.635	0.1634	95.65	38.89	0.345	≤194.6 pg/mL
IL-6	0.592	0.3111	26.09	100.00	0.261	>18.16 pg/mL
Uric acid	0.639	0.1213	78.26	55.56	0.338	≤156.78 µmol/L
Subjects with normal weight—prediction of abnormal LH/FSH ratio						
Salivary testosterone	0.811	**0.0044**	100.00	60.00	0.600	>32.6 pg/mL
TNF-α	0.600	0.4992	44.44	90.00	0.344	>17.29 pg/mL
IL-1β	0.639	0.3567	100.00	50.00	0.500	≤183.2 pg/mL
IL-6	0.733	0.0614	55.56	90.00	0.456	>10.92 pg/mL
Uric acid	0.617	0.3946	88.89	40.00	0.289	≤199.14 µmol/L
All PCOS subjects—prediction of too high FAI						
Salivary testosterone	0.658	0.0749	52.63	81.82	0.345	>45.35 pg/mL
TNF-α	0.730	**0.0042**	73.68	68.18	0.419	>16.52 pg/mL
IL-1β	0.597	0.2912	84.21	50.00	0.342	≤182.78 pg/mL
IL-6	0.711	**0.0104**	63.16	72.73	0.359	>9.58 pg/mL
Uric acid	0.537	0.6911	73.68	45.45	0.191	≤156.78 µmol/L
Subjects with normal weight—prediction of too high FAI						
Salivary testosterone	0.671	0.2090	100.00	42.86	0.429	>32.63 pg/mL
TNF-α	0.657	0.3530	60.00	85.71	0.460	>17.27 pg/mL
IL-1β	0.629	0.3206	100.00	50.00	0.500	≤182.78 pg/mL
IL-6	0.700	0.2697	60.00	92.86	0.529	>11.18 pg/mL
Uric acid	0.593	0.5151	100.00	35.71	0.357	>127.51 µmol/L
All PCOS subjects—prediction of higher cortisol/DHEA-S ratio						
Salivary testosterone	0.543	0.6490	80.95	45.00	0.260	≤47.52 pg/mL
TNF-α	0.544	0.6357	71.43	50.00	0.214	≤17.27 pg/ mL
IL-1β	0.629	0.1547	85.71	40.00	0.257	≤185.09 pg/ mL
IL-6	0.587	0.3417	33.33	90.00	0.233	>14.08 pg/ mL
Uric acid	0.698	**0.0223**	57.14	85.00	0.421	>156.78 µmol/L
Subjects with normal weight—prediction of high cortisol/DHEA-S ratio						
Salivary testosterone	0.667	0.2166	80.00	66.67	0.467	≤40.61 pg/ mL
TNF-α	0.522	0.8778	40.00	88.89	0.289	≤14.75 pg/ mL
IL-1β	0.600	0.4713	90.00	33.33	0.233	≤185.09 pg/ mL
IL-6	0.589	0.5332	70.00	55.56	0.256	>8.90 pg/ mL
Uric acid	0.828	**0.0030**	80.00	88.89	0.689	>149.57 µmol/L
All PCOS subjects—prediction of too high estradiol level						
Salivary testosterone	0.811	**<0.0001**	100.00	69.44	0.694	>44.40 pg/ mL
TNF-α	0.600	0.4224	100.00	30.56	0.306	>14.86 pg/ mL
IL-1β	0.506	0.9684	80.00	47.22	0.272	>169.83 pg/ mL
IL-6	0.614	0.4981	80.00	61.11	0.411	>9.59 pg/ mL
Uric acid	0.597	0.3368	100.00	44.44	0.444	>129.18 µmol/L
Subjects with normal weight—prediction of too high estradiol level—N.A. (number of positive cases = 1)						

ROC—receiver operating characteristic, AUC—area under the curve, BMI—body mass index, TNF-α—tumor necrosis factor α, IL-6—interleukin 6, IL-1β—interleukin 1β, FAI—free androgen index, DHEA-S—dehydroepiandrosterone sulfate, LH—luteinizing hormone, FSH—follicle-stimulating hormone, statistically significant *p*-values in bold.

## Data Availability

The datasets are not publicly available due to privacy restrictions but can be obtained from the corresponding author upon reasonable request.
